# A Retrospective Review of a Bed-mounted Projection System for Managing Pediatric Preoperative Anxiety

**DOI:** 10.1097/pq9.0000000000000087

**Published:** 2018-06-22

**Authors:** Thomas J. Caruso, Jeremy H. Tsui, Ellen Wang, David Scheinker, Paul J. Sharek, Christine Cunningham, Samuel T. Rodriguez

**Affiliations:** From the * Department of Anesthesiology, Perioperative and Pain Medicine, Stanford University School of Medicine, Stanford, Calif.; † Department of Management Science and Engineering, Stanford University, Stanford, Calif.; ‡ Department of Perioperative Services, Lucile Packard Children’s Hospital, Palo Alto, Calif.; § Division of Hospitalist Medicine, Department of Pediatrics, Stanford University School of Medicine, Stanford, Calif.; ¶ Center for Quality and Clinical Effectiveness, Lucile Packard Children’s Hospital, Palo Alto, Calif.; ‖ Office of Patient Experience, Lucile Packard Children’s Hospital, Palo Alto, Calif.

## Abstract

**Introduction:**

Most children undergoing anesthesia experience significant preoperative anxiety. We developed a bedside entertainment and relaxation theater (BERT) as an alternative to midazolam for appropriate patients undergoing anesthesia. The primary aim of this study was to determine if BERT was as effective as midazolam in producing cooperative patients at anesthesia induction. Secondary aims reviewed patient emotion and timeliness of BERT utilization.

**Methods:**

We conducted a retrospective cohort study of pediatric patients undergoing anesthesia at Lucile Packard Children’s Hospital Stanford between February 1, 2016, and October 1, 2016. Logistic regression compared induction cooperation between groups. Multinomial logistic regression compared patients’ emotion at induction. Ordinary least squares regression compared preoperative time.

**Results:**

Of the 686 eligible patients, 163 were in the BERT group and 150 in the midazolam. Ninety-three percentage of study patients (290/313) were cooperative at induction, and the BERT group were less likely to be cooperative (*P* = 0.04). The BERT group was more likely to be “playful” compared with “sedated” (*P* < 0.001). There was a reduction of 14.7 minutes in preoperative patient readiness associated with BERT (*P* = 0.001).

**Conclusions:**

Although most patients were cooperative for induction in both groups, the midazolam group was more cooperative. The BERT reduced the preinduction time and was associated with an increase in patients feeling “playful.”

## INTRODUCTION

Most children undergoing anesthesia experience significant preoperative anxiety.^1–4^ Alleviation of preoperative anxiety is an important component of pediatric perioperative management because it has been associated with increased postoperative pain, emergence agitation, and sleep disturbances.^5,6^ One common approach to preoperative anxiolysis is through the administration of oral benzodiazepines such as midazolam. Oral midazolam has an onset time of 15 minutes and may improve parental separation anxiety and cooperation with the anesthesiologist.^7–9^ However, patient noncompliance during midazolam administration is common due to midazolam’s unpleasant taste, and it can sometimes lead to a paradoxical reaction resulting in hyperactivity.^10,11^

Given the recent Food and Drug Administration warning to limit anesthetics in children, nonpharmacological anxiolytics have gained popularity as an alternative to pharmacological agents.^12–15^ These include parental-present inductions and technology-based distraction devices such as tablets, phones, video games, and video glasses.^15–18^ Despite reports of effectiveness, they share limitations including time, cost, and need for child cooperation.

Recently, we reported the development of a low-cost bedside entertainment and relaxation theater (BERT) as an alternative method of preoperative anxiolysis.^19^ We hypothesized that the near-immersive, large, bed-mounted screen coupled with its novelty would reduce preoperative anxiety. The primary aim of this retrospective study was to determine the effectiveness of BERT compared with oral midazolam as a preoperative anxiolytic, as measured by cooperation during induction. The 2 secondary aims were to (1) explore differences in the emotional state of patients during induction and (2) determine time differences between BERT and midazolam utilization.

## METHODS

### Context and Study Population

After institutional ethics review board granted a research waiver (Stanford University, Calif.), a retrospective cohort study was conducted at a freestanding, 311-bed academic pediatric hospital in Northern California. There are 8 preoperative beds, 7 operating rooms (ORs), and 13 out-of-OR anesthetizing locations (such as ambulatory procedure suites). There are 2 child life specialists who work in the preoperative area. Medical records for all patients receiving anesthesia between February 1, 2016, and October 1, 2016, were reviewed.

There were 2 groups studied: the BERT group (included patients who utilized the BERT system and had a parental-present induction) and the midazolam group (included patients who received oral midazolam and did not have parental-present induction). Both groups underwent inhalation induction. Selection of patients into the midazolam or BERT groups was dependent upon provider and patient preference. At the time of arrival, patients and families were offered several anxiolytics, including child life consultation, parent-presence induction, tablet or phone distraction therapy, or BERT. Although providers were generally amenable to patient preference, in certain situations, such as induction of patients with known difficult airways, providers typically would not allow parent-present inductions. Exclusion criteria were (1) patients who received cardiac anesthesia care; (2) those receiving anesthesia at out-of-OR locations where BERT was not offered; (3) those who received multiple anxiolytics (such as both BERT and midazolam); and (4) those with anesthesia charts lacking the measures for this review.

### Intervention

**Fig. 1.**
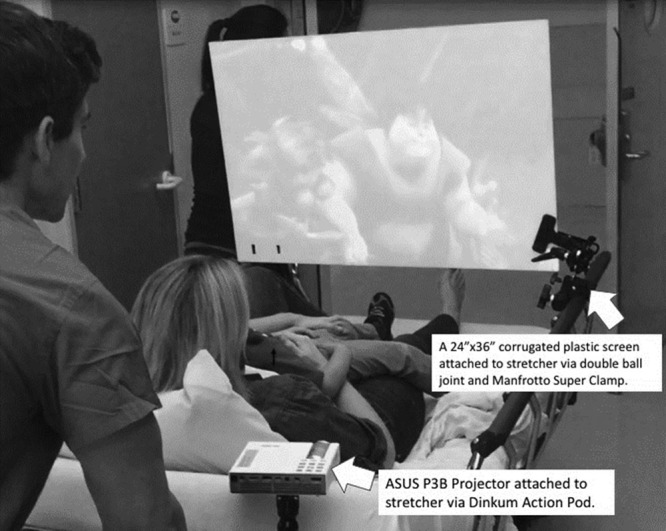
BERT setup.

BERT consists of a portable, battery-powered Asus P3B projector (AsusTek Taipei, Taiwan) enclosed in a custom Plexiglas case (approved by institution Infection Prevention) and mounted with a Dinkum ActionPod clamp (DinkumSystems, Boulder, Colo.) at the head of the patient’s bed. At the foot of the bed is a 24 × 36 inch sealed corrugated plastic screen mounted with a modified Manfrotto Super Clamp (Manfrotto, Upper Saddle River, N.J.) (Fig. 1).^19^ The BERT system costs approximately $750. The patients have a choice of age-appropriate videos that are started in the preoperative area and continued until the child is under anesthesia in the OR.

### Measures

Data were collected via chart review of electronic medical records (EMR) using EPIC (Epic Systems Corporation, Verona, Wis.). Demographic data collected included sex, age, and American Society of Anesthesiologists (ASA) status.

### Primary Aim: Effectiveness of BERT

The effectiveness of BERT compared with midazolam was determined by analyzing whether the patient was cooperative at induction. These data were collected from the anesthesia induction note that relied on anesthesiologists’ judgment to record induction cooperation as a dichotomous choice (yes or no). Recording patients’ cooperation with induction was a preexisting component of the EMR before initiation of this retrospective review.

We assumed the overall mean success rates of midazolam to be 75%.^20,21^ We defined any difference within 15% to be of no clinical importance. With the noninferiority margin being selected to be -0.15, a required sample size with equal allocation was estimated to be 152 for each group to achieve a power of 80% with a 2-sided significance level of 0.05. Patient records were reviewed retrospectively until the sample size was met to ensure adequate power.

### Secondary Aim 1: Emotional State of Patients at Induction

The emotional state of patients was determined by anesthesiologists at the time of induction. The choices included medically sedated, age-appropriate, playful, reserved, anxious, distressed, and panicked. These emotional states were a preexisting component of the anesthesia induction note and are used by anesthesiologists and child life specialists to gauge the effectiveness of anxiolytic interventions.

### Secondary Aim 2: Timeliness of BERT Utilization

Timeliness of the BERT intervention was measured by comparing the time of patient readiness for anesthesiologist consultation in the preoperative area until induction of anesthesia. These data were obtained by reviewing EMR timestamps that are recorded by preoperative and OR nurses as patients progress through the perioperative process.

### Data Analysis

#### Primary Aim: Effectiveness of BERT.

Unpaired *t* tests were used to determine group differences with regard to age, sex, and ASA score. Logistic regression was used to compare the relative risk of a patient being uncooperative in the BERT group compared with the midazolam group while controlling for patient age, ASA score, and sex. Results were considered significant at a *P* value less than 0.05. R (version 3.4.1, Boston, MA, USA) and RStudio (version 1.0.153, Boston, MA, USA) were used to conduct the analysis.

#### Secondary Aim 1: Emotional State of Patients at Induction.

The incidence of emotional states was reported. If an emotional state had < 5% incidence, it was grouped into an “other” category. Multinomial logistic regression was used to compare patients’ likelihood of being “playful,” “medically sedated,” “age-appropriate,” and “other” at induction in the BERT compared with the midazolam group.

#### Secondary Aim 2: Timeliness of BERT Utilization.

Ordinary least squares regression was used to compare the time between “patient ready to be seen by an anesthesiologist” in the preoperative area and “induction” in the OR for the 2 groups while controlling for age, sex, and ASA score.

## RESULTS

**Fig. 2.**
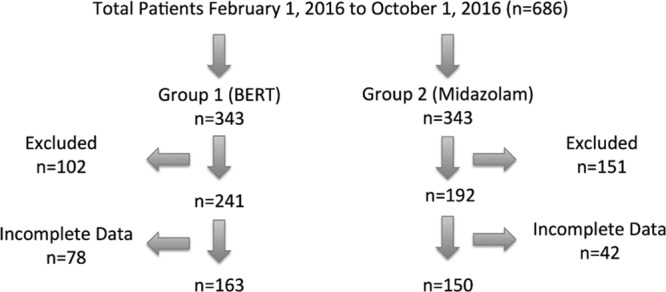
Enrollment.

Six hundred eighty-six patients were reviewed during the study period. Of these, 343 patients were in the BERT group and 343 patients in the midazolam group. A total of 264 patients were excluded as a result of overlapping interventions such as a patient receiving both BERT and midazolam, or midazolam with other pharmacological agents. An additional 120 patients were excluded due to missing data, resulting in a study population of 163 patients in the BERT group and 150 patients in the midazolam group (Fig. 2).

### Demographics

There were no differences between sex and age (Table 1). There was a higher proportion of ASA I and II patients in the BERT group and a lower proportion of ASA III and IV patients in the BERT group (Table 1).

**Table 1.**
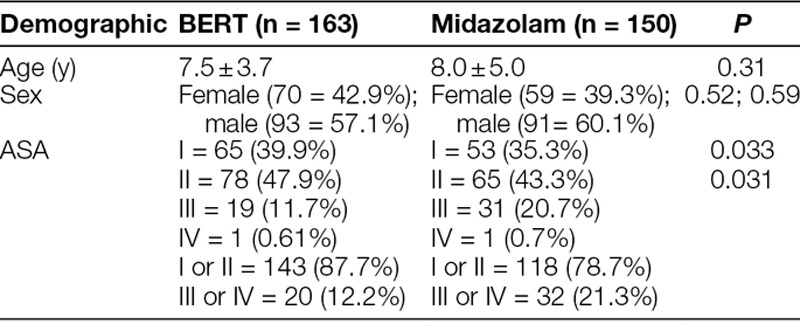
Demographics

### Primary Aim: Effectiveness of BERT

Ninety-three percentage of all study patients (290/313) were cooperative at induction. Patients in the BERT group were less likely to be rated “cooperative” at induction compared with patients in the midazolam group when controlling for patient age, ASA score, and sex (odds ratio = 0.37, *P*-value = 0.043).

### Secondary Aim 1: Emotional State of Patients at Induction

The total number of patients with each emotional states as reported by anesthesiologists at induction was as follows, reported as (BERT group, midazolam group): medically sedated (3, 64); age-appropriate (74, 55); playful (66, 19); reserved (11, 8); anxious (5, 3); distressed (1, 1); and panicked (3,0). Reserved, anxious, distressed, and panicked had an incidence of < 5% and were therefore analyzed in the “other” category. Patients in the BERT group were significantly more likely to be rated “playful,” “age-appropriate,” or “other,” compared with “medically sedated” in the midazolam group when controlling for patient age, ASA score, and sex [multinomial relative risk = 10.88, *P* value < 0.001 (playful); 2.51, *P* value < 0.001 (age appropriate); 3.41, *P* value < 0.001 (other)].

### Secondary Aim 2: Timeliness of BERT Utilization

There was a reduction of 14.7 minutes in the time between “patient ready to be seen by an anesthesiologist” in the preoperative area to “induction” in the OR associated with the BERT group compared with the midazolam group when controlling for age, sex, and ASA (*P* = 0.0014).

## DISCUSSION

This study is the first that examines the effectiveness of a bedside projection system compared with midazolam. Although the majority of patients in both groups were cooperative at induction, the BERT group was associated with less induction cooperation when compared with the midazolam group. Despite this minor difference in lack of cooperation, given midazolam’s side effects, which include nausea, dizziness, and agitation, utilization of nonmedicinal anxiolytics such as BERT should be considered.^12,14^ Additionally, because oral midazolam requires up to 15 minutes to take effect, utilization of BERT was timelier than midazolam.^9^

Given the Food and Drug Administration’s warning to limit anesthetics in children, we developed BERT as an alternative nonmedicinal tool for preinduction anxiolysis.^12^ BERT’s novelty provides an advantage over conventional handheld devices that have become a common part of the culture. The large screen size of BERT surprises and captures the patients’ attention, which is consistent with studies that have reported an association between size and preference in images.^22^ Because BERT travels into the OR, the patient’s attention is uninterrupted even as the anesthesiologist performs an inhalational induction. BERT successfully transformed anesthesia induction into a playful experience. Children who require multiple medical exposures are more likely to develop anxiety and fear of health care providers.^23^ The development of methods to convert medical exposures from fearful to playful experiences may reduce the risk of pediatric stress.

To integrate BERT into our perioperative workflow, we engaged our perioperative improvement team, which consists of frontline anesthesiologists, surgeons, child life specialists, nurses, patient experience officers, pharmacists, and quality improvement managers. The perioperative team meets weekly to discuss and implement projects aimed at improving patient care. Given the diminishing effectiveness of handheld technological devices, the integration of this novel bedside projector system into standard perioperative work was predicted to reduce midazolam use and increase efficiency. After training the preoperative care team (nurses, nurse practitioners, child life specialists, and anesthesiologists) on BERT’s functionality, implementation was initiated. Any member of the preoperative care team was capable of identifying a potential candidate for BERT and preparing the BERT system on the gurney. The clamps, screens, and projectors were strategically located in a cabinet in the preoperative area to enhance accessibility. After the patient was induced, BERT was placed on the patient’s gurney outside of the OR where OR assistants cleaned and returned the system to the preoperative area. By incorporating the frontline staff into the development of the process, we were able to ensure buy-in.^24^

This study had several limitations. First, as with any retrospective study, there may have been unmeasured biases, such as stylistic differences between anesthesiologists, which affected the anxiolytic choices offered to patients. Depending on the multiple patient and provider factors and implicit biases, calmer children may have been offered BERT or midazolam, altering the results in an unknown direction. Second, this study relied on anesthesiologist reported descriptions of cooperation and emotional state at the time of induction. Inter-rater reliability may have been reduced, given the subjective nature of the assessments and the lack of a validated tool for measuring these outcomes. However, given the large, equal sample sizes, we have no reason to believe that the assessments would have favored 1 group. Finally, the BERT group included parental-presence inductions, unlike the midazolam group, because at our institution, patients who did not receive a pharmacologic anxiolytic were routinely offered a parental-present induction. The lack of cooperation with induction in the BERT group may have been attributed to the parental-presence. Previous studies have failed to show a significant difference with parental-presence on child anxiety consistently, and some studies have demonstrated an increase in anxiety with parental-present inductions, especially with anxious parents.^25–27^

Using a bedside mounted projector should be considered for nonpharmacologic preoperative anxiolysis. It is low-cost, novel, and increases the efficiency of preoperative patient preparation with minimal impact on patient cooperation. Prospective, randomly designed studies are needed to confirm the effectiveness of BERT and determine which patients are optimally suited for BERT.

## ACKNOWLEDGMENTS

Assistance with study: The authors thank Dr. Ban C. H. Tsui, Department of Anesthesia, Stanford University, for his guidance and contributions and the Traverse Foundation.

## DISCLOSURE

The authors have no financial interest to declare in relation to the content of this article.
